# Combining preclinical tools and models to unravel tumor complexity: Jump into the next dimension

**DOI:** 10.3389/fimmu.2023.1171141

**Published:** 2023-03-24

**Authors:** Giacomo Miserocchi, Martine Bocchini, Michela Cortesi, Chiara Arienti, Alessandro De Vita, Chiara Liverani, Laura Mercatali, Sara Bravaccini, Paola Ulivi, Michele Zanoni

**Affiliations:** ^1^ Biosciences Laboratory, IRCCS Istituto Romagnolo per lo Studio dei Tumori (IRST) “Dino Amadori”, Meldola, Italy; ^2^ Osteoncology and Rare Tumors Center, Immunotherapy, Cell Therapy and Biobank, IRCCS Istituto Romagnolo per lo Studio dei Tumori (IRST) “Dino Amadori”, Meldola, Italy

**Keywords:** cancer, 3D models, tumor microenvironment, zebrafish, organoids

## Abstract

Tumors are complex and heterogeneous diseases characterized by an intricate milieu and dynamically in connection with surrounding and distant tissues. In the last decades, great efforts have been made to develop novel preclinical models able to recapitulate the original features of tumors. However, the development of an *in vitro* functional and realistic tumor organ is still utopic and represents one of the major challenges to reproduce the architecture of the tumor ecosystem. A strategy to decrypt the whole picture and predict its behavior could be started from the validation of simplified biomimetic systems and then proceed with their integration. Variables such as the cellular and acellular composition of tumor microenvironment (TME) and its spatio-temporal distribution have to be considered in order to respect the dynamic evolution of the oncologic disease. In this perspective, we aim to explore the currently available strategies to improve and integrate *in vitro* and *in vivo* models, such as three-dimensional (3D) cultures, organoids, and zebrafish, in order to better understand the disease biology and improve the therapeutic approaches.

## 1 Introduction

**Figure 1:**
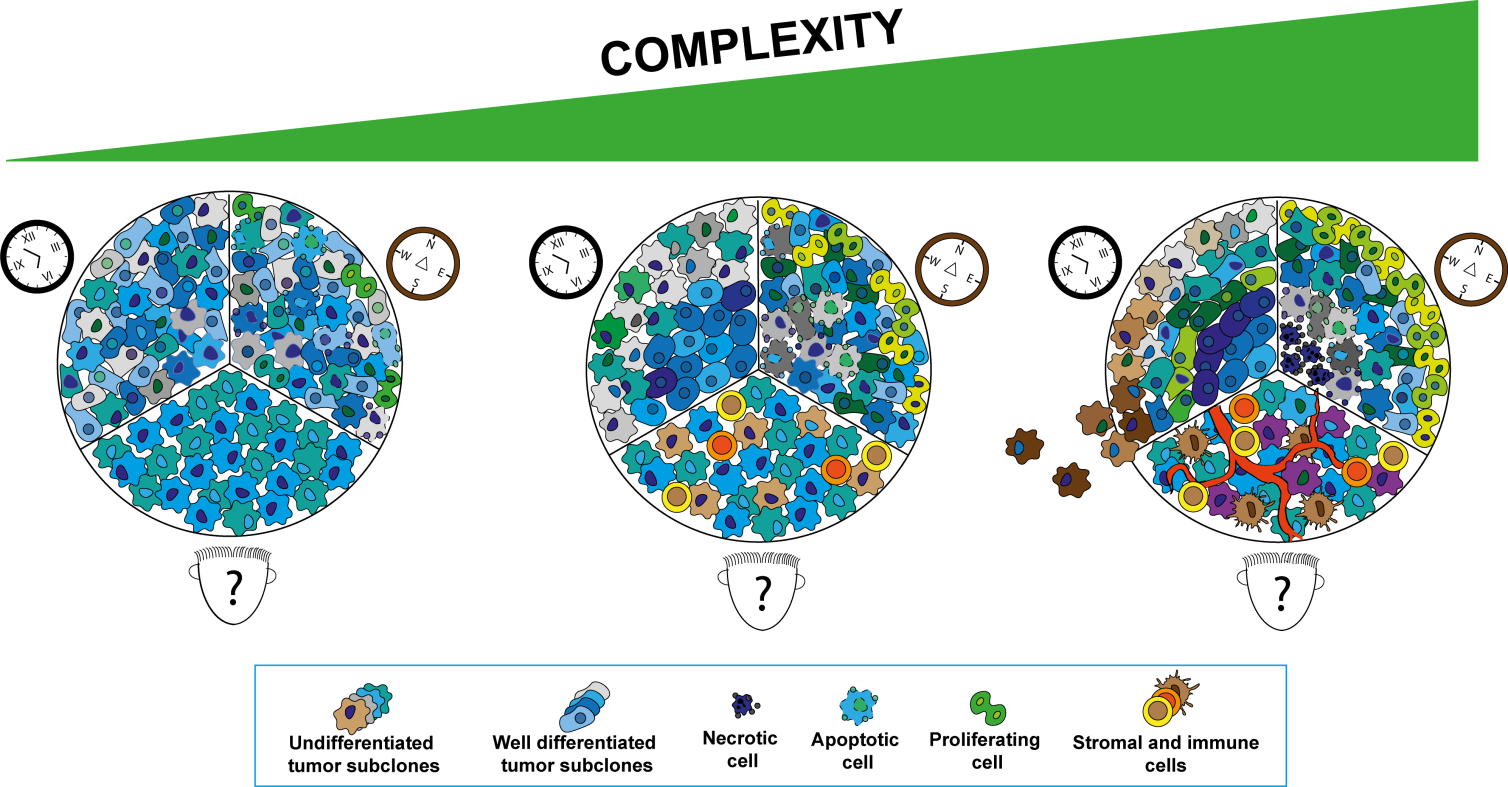
Schematic representation of the tumor development aspects to be consider in cancer modeling subdivided in three main aspects. “Who?” cellular populations composing the tumor microenvironment (TME) (human face cartoon), “Where?” spatial distribution of the different cellular components in the TME (compass cartoon) and “When?” tumor development at different stages (clock cartoon). Increase in complexity in tumor modeling can be reached progressively adding different tumor sub-clones, stromal and immune cells in the system at the ratio reported in the tumor subtypes of interest. The original spatial distribution of cellular components in tumor tissues can be mimicked by reconstructing regions characterized by different biochemical gradients (i.e., oxygen, metabolites) and physical properties. The different stages of neoplastic transformation can be reproduced by systems able to recapitulate the tumor initiation and progression processes closely recapitulating the oncogenic development.

Cancer is a complex disease characterized by the sequential accumulation of genetic alterations in a progressive and dynamic process deeply influenced by the surrounding environment (1, 2). Tumors are not simply an assembly of cancer cells but rather they operate as “abnormal organs” that evolve constantly interacting with the microenvironment, exploiting peculiar processes of tissue remodeling and organs development (3). Given that, knowing individual tumor components is necessary but perhaps insufficient to understand the behavior of the whole system (2). In the last decades, researcher’s efforts aimed to face off such complexity by developing cancer models that closely recapitulate most of the aspects of the oncologic disease in order to understand its pathophysiological features and to design novel and more effective therapeutic strategies (**Figure 1**). Determining the composition of the tumor microenvironment, as well as knowing if, how and at what stage of development, tumor cells interact with each other and with immune and stromal cells in surrounding and distant tissues is crucial to improve patient’s outcome. The advent of single cell genomic and spatial profiling technologies have improved our ability to dissect cancer networks, allowing to define the identity of cells and their function in the native environment of complex tissue samples (4). This knowledge, translated in tissue bioengineering, offers a unique opportunity to generate more reliable and sophisticated 3D cancer models. To date, combining, for example, microfluidic systems with advanced 3D-bioprinting technologies represent an extremely powerful tool that allowed to reproduce key pathophysiological, physical, and biochemical cues found *in vivo* in a temporal and spatially controlled modality, whilst providing a dynamic and “systemic” tissue environment (1). However, in vivo models still represent an essential tool to study molecular mechanisms of tumorigenesis, cancer progression and dissemination as well as novel therapeutic strategies before proceed with clinical trials. Several different animal models are currently in use for cancer research and researcher can choose based on animal’s characteristics and on the research question to be addressed. The combination of in vitro 3D models with *in vivo* models might take preclinical research to the next step allowing to perform more robust and reliable research and, in turn, to develop more effective anti-cancer treatments. Herein we will discuss the currently available strategies to improve and integrate *in vitro* and *in vivo* tumor models, in order to better understand the biology of the disease and to facilitate the transfer of bench side discoveries to patient’s bedside.

## 2 Cellular composition (Who)?

The tumor microenvironment (TME) is a complex network encompassing several cellular entities, including immune cells and non-cellular components such as the extracellular matrix, exosomes and interleukins (5). These cellular and extracellular entities are defined to take part in cancer development, progression and immune system exhaustion (6). Today, wide varieties of *in vivo* models are used to reconstruct primary and metastatic tumors niche and microenvironment (7). Human malignant components interact with the host cell populations through a biochemical dialog between cancer cells and surrounding tissues, an aspect that must be considered due the high number of variables involved. Conversely, current *in vitro* models using immortalized cell lines, rather than primary cells, fail to recapitulate many of the known features of tumors *in vivo*. On the other hand, cell populations obtained from biopsies or resected tumor sections are difficult to characterize and define; a disadvantage also belonging to *in vivo* systems. In this context, using more controllable and well-characterized models leads to reduce the number of undesired experimental bias.

**Figure 2:**
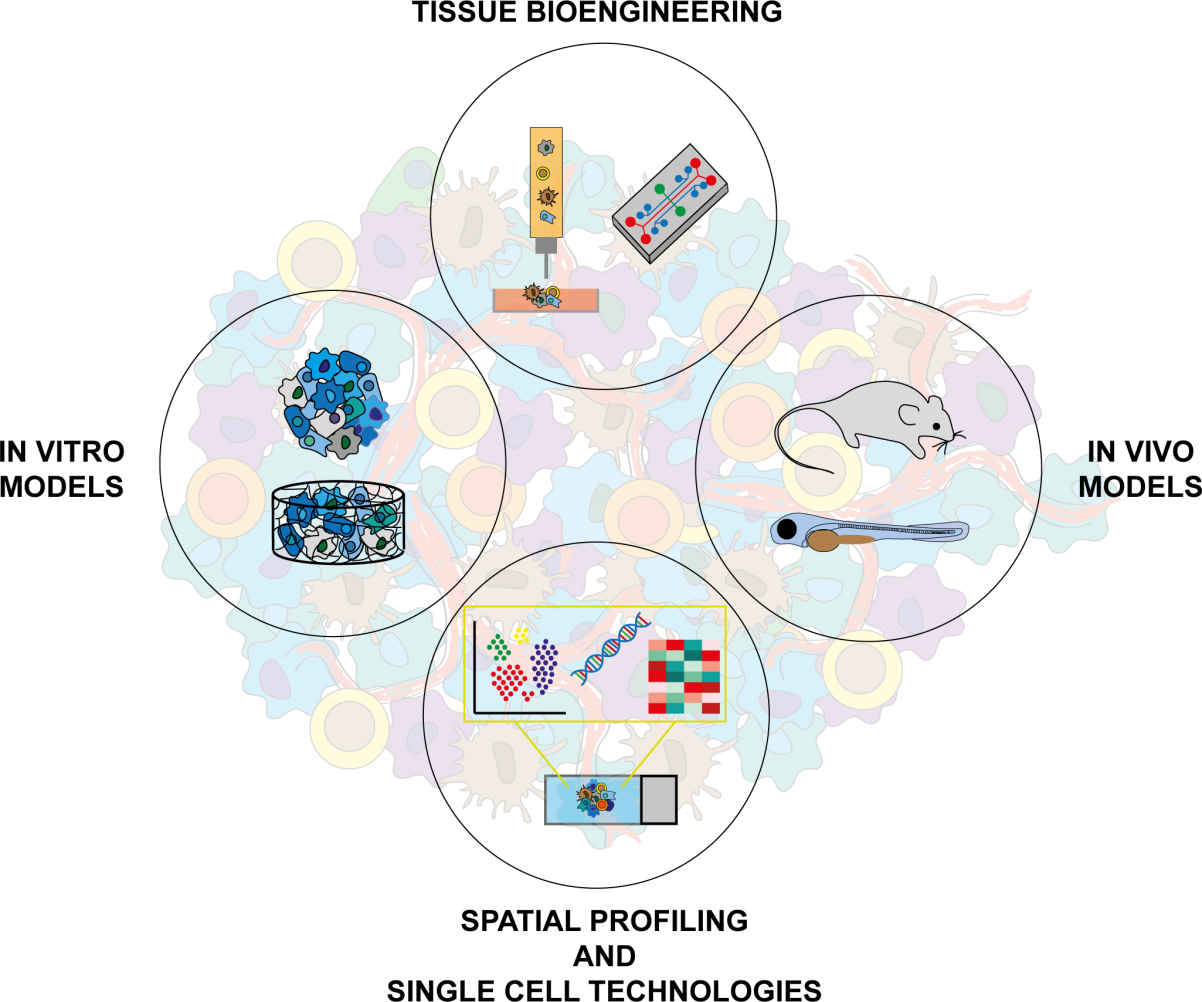
Integration of different approaches and technologies is necessary to increase complexity in cancer modeling. *In vitro* models: 2D cultures, biomimetic 3D scaffold cultures, spheroids, organoids. Tissue bioengineering: bioprinting technologies, bioreactors. *In vivo* models: GEMMs, embryos and adult zebrafish. Spatial profiling and single cell technologies: digital spatial profile techniques, single cell sequencing.

In this context, the selection of the best actors represents one of the first steps. Research groups can take advantages from single-cell omic approaches to map the TME cell heterogeneity. In particular, different cellular populations and their respective transcriptomic and genomic profiles can be detected at single-cell level allowing to define both sample composition and cellular evolutionary relationships (8). To obtain the genomic profile of each single element, the sample preparation needs an initial step of single cell isolation followed by extraction and amplification of the genetic material. Then, bioinformatics analysis are used to cluster the omic profiles in the different cellular subpopulations (9). These multi-omics analysis represented a crucial step forward to dissect TME and, thus, for the generation of more realistic *in vitro* tumor models. Over the past few years, growing efforts have been put into the development of 3D *in vitro* models in which tumor cells are cocultured with a variety of stromal cells (10). This approach allows reproducing selected cell-cell and stromal-cell interactions. Recent studies have shown how synthetic or patient-derived 3D platforms can support the development of spheroid tri-cultures cancer models as a system to assess drug responses and oncogenic processes (11, 12). The physical and molecular crosstalk between cancer and multiple stromal cells within different 3D devices, spheroids and tissue-like structures, were used to resemble the TME complexity. The integration of multiple cell types in a single culture provides an accurate mimicking of the TME, but the maintenance of different subpopulations in an *in vitro* environment needs to be finely tuned in order to avoid phenotype modification toward non-physiological setting, for the culture medium, duration and overall (13). Up to now, one of the main criticisms of developing comprehensive models is represented by the incorporation of immune cells into a 3D culture. Protocols for this kind of implementation include immune cells penetration into mature spheroids, microfluidics technologies as a purpose for simulating the dynamic cancer environment and immune cells bioprinting as a purpose for allowing the correct deposition and alignments of immune population on scaffold-based models and organoids (14–16). In this field, simple co-culture of 3D systems with immune populations were used to test their role on the effect of immunotherapies. For this reason, Courau T. et al. showed how two co-culture approaches, in particular allogeneic T and NK cells with colorectal cancer cell line-derived spheroids and autologous tumor-infiltrating lymphocytes with patients-derived organoids, can represent valid tools to study the antitumor potential of immunomodulatory antibodies (17). The use of multicellular organoids derived from minced tumor explants allows the conservation of the spatial distribution and architecture of the tissue, all aspects lost by enzymatic digestion processes. To further increase the complexity of the model, different groups demonstrate how the direct transplantation of patient’s tissue fragments in zebrafish (ZF) embryos, named ZF “Avatars”, can reflect the patient’s outcome and could support co-clinical trials (18, 19). It represents an additional step towards studies of precision medicine, but it seems unlikely to be applied to preclinical projects due the complex sample’s characterization and the limited amount of material. In this context, the transplantation of more controllable 3D models could integrate the advantages of a simple *in vivo* system and could preserve the “spatio-temporal features”of tissue-mimetic cultures, all steps to gradually reach a reliable *in vitro*/*in vivo* cancer organ (**Figure 2**).

## 3 Spatial distribution (Where)?

Reproducing the spatial distribution of cancer cellular and acellular components represents a challenging aim for *in vitro* models. Malignant tissues are characterized by alterations in the natural structure and density of extracellular matrix (ECM), features that play crucial roles in the modulations of pathophysiological processes (20, 21). So, the preservation of the tumor niche architecture and protein composition acquires a remarkable value. “Patients-derived” scaffolds obtained by tissue decellularization techniques represent probably one of the most effective options in this field, but their applications are limited due the narrow amount of material (22). The possibility to synthetize biomimetic scaffolds, designed on the personal protein composition of patients ECM, allows to partially overcome this limitation. The identification of specific ECM components remains difficult to achieve due to the biochemical characteristics of matrix structures (23). Matrisome analysis, a combination of in silico and proteomic techniques, represent a valid system to explore the ECM composition. Below et al. used this kind of approach to optimize the synthesis of PEG hydrogels in order to supply a realistic *in vitro* TME (24). The model was able to reproduce the stiffness range of human and murine pancreatic tumors and to display the expression by different co-cultured stromal cell populations of genes associated to tumor-stromal interactions. This approach provides a defined pattern and reproducible system to study the cells/TME interactions considering the contribution of the specific ECM elements.

Spatial-profiling technologies represents another novel and useful tool that can support accurate TME 3D modelling for the design of biomimetic representative *in vitro* tumor models. Unlike most common bulk or single cells technologies that operate on cell dissociated from the tissues of interest, this approach allow identifying at transcriptomic and genomic level, the cellular components and their function in the native spatial context of the tumor tissue (4). In addition, these technologies enable to select specific area of interest in the tumor tissues through multiple antibody-based and probe-based assays; offering the unique opportunity to evaluate expression profiles at single-cell resolution of isolated tumor/stromal/immune cells or when interacting. Indeed, the seeding of cell suspensions inside a scaffold area produces a random cell disposition that is difficult to control. Bioprinting technologies are innovative approaches that allow us to observe the distribution of ECM components, cell populations and biomolecules (25, 26). Immortalized cell lines are the most employed bioinks, but recently, remarkable advances were performed to include also primary samples. One example is represented by the complex human-glioblastoma-on-a-chip developed by Dong-Woo Cho’s group (27). This model uses patient-derived tumor cells, porcine brain tissue-derived ECM and vascular endothelial cells organized in a compartmentalized structure for the reproductive purpose of the biochemical-physical properties of the native cancers. This system displayed a concentric distribution of proliferative, hypoxic, and necrotic areas able to recreate the pathological features of the tumor and replicate the patient-specific drug response. In the era of personalized medicine, the implementation of autologous systems is unavoidable but we must also consider their reproducibility. The synthesis of controlled devices based on the study of tissue composition could represent a promising approach to obtain a realistic *in vitro* TME reducing the employment of limited primary samples and increasing the number of feasible analyses.

## 4 Temporal development (When)?

Cancer is extremely heterogeneous and constantly changes its own structure and composition during all the pathological stages. This process mainly affects the tumor niche inducing drastic alterations in surrounding and distant tissues (28). Reproducing such aspects in tumor models is essential to have a comprehensive view of the oncological disease, from the initial steps of neoplastic transformation to metastatic dissemination, and to test specific treatments at the most appropriate time. Modeling the different events involved in neoplastic evolution remains challenging, mainly due to the difficulty of reproducing *in vitro* the physiological condition described *in vivo*. Indeed, *in vivo* models could represent the most realistic option where cancer cells are exposed to all the biochemical, physical and physiological stimuli of a complete organism, a characteristic ideal to obtain the final translational data but that does not discriminate the influence of the single actors. Genetically engineered mouse models (GEMMs) represent a suitable model to study early neoplastic transformation *in vivo* allowing the development of tumors *de novo* in an immune-proficient microenvironment (29). However, these models do not always recapitulate pathophysiological features of human tumors, and are expensive and time-consuming to develop (30, 31). In this context, *in vitro* approaches offer the possibility to focus the attention on fewer processes in more controllable systems. Indeed, organoids represent a powerful and robust model that can be used for tissue-specific modeling of tumor initiation and progression closely recapitulating the oncogenic process (32). Several studies reported the use of CRISPR/Cas9-based genome systems to introduce multiple mutations into organoids derived from different normal human epithelia leading to their neoplastic transformation (33–35). In addition, organoids can be employed to study tumorigenesis processes triggered by cancer-related environmental factors, such as oncogenic pathogens. Indeed, in a recent paper by Pleguezuelos-Manzano et al, it has been demonstrated that the prolonged exposure of human intestinal organoids to genotoxic Escherichia coli directly causes mutations in host epithelial cells (36). Established tumors display a structural and functional architecture characterized by areas exposed to an impaired distribution of nutrients and oxygen and consequently composed by cells with altered phenotypic, physical, functional, and metabolic features. This has a substantial impact on the behavior of both cancer and stromal cells, resulting in a heterogeneous response to the different treatments. Organoids as well as spheroids and other scaffold-based systems can only mimic some of these tumor features but still lack the multidimensionality and spatio-temporal dynamics of native tumors. However, generate reliable bio-and physio-mimetic models that take into account all fundamental tumor building hallmarks such as hypoxic/metabolic niches, blood/lymphatic vasculature, structural architecture, multicellular heterogeneity, ECM biochemical composition and mechanical properties, as well as tumor-specific fluid dynamics is still far from being completely reached. 3D bioprinted cancer-on-a-chip models represented the most innovative microfluidic devices able to recapitulate tumor structure providing also a multi organ-level dimension under dynamic fluid flow (1). Various biomimetic materials can be combined with tumor and stromal cells in a well-defined spatially controlled cellular organization.

Based on this idea, Heinrich and colleagues used 3D-bioprinting to fabricate 3D mini-brain models of glioblastoma (GBM), combining cancer cells and macrophages in a more structural and anatomical scale with the aim to study the crosstalk between macrophages and cancer cells in terms of proliferation, migration, and resistance to therapy (37). A step further has been done by Cao and colleagues that fabricate a bioprinted tumor-on-a-chip device which include vascular and lymphatic perfusable vessels allowing of simulating the transport mechanisms of biomolecules and anticancer drugs inside the tumor microenvironment (38). This approach can be also used to mimic the metastatic dissemination processes typical of the late stages of the disease. Separated chambers in continuous interconnection host different kinds of cell cultures, from live cells to entire tissues, to simulate *in vitro* organs. For instance, the organs-on-a-chip of Skardal et al. mimicked the metastasization processes of colon carcinoma cells from hydrogel-based gut constructs to liver tissue cells (39). The choice of hydrogel cultures allows the regulation of the structure stiffness through the administration of crosslinking agents in order to explore the involvement of the physical microenvironment parameters in the metastatic processes. The reliability of these systems has been widely demonstrated up to speculate their application as support to clinical trials. In this context, Chramiec A. et al. designed a microfluidic platform based on the interconnection of two bioengineered tissues, bone Ewing Sarcoma tumor and heart muscle, to test efficacy and cardiotoxicity of oncologic treatments (40). The combination of 3D hydrogel, decellularized bone scaffold, and a microfluidic platform, three translational models highly characterized, has allowed the development of a more complex system able to reproduce the results of a clinical trial. This clear synergy between multi-organ on-chip systems and innovative culture models suggests a promising future for the application of these kinds of platforms as patient’s avatars for the design of personalized treatments. Moreover, the possibility to adopt “humanized” and easily controllable devices could decrease the employment of animals through their replacement with artificial systems. However, *in vivo* models still represent the most realistic “patient simulators” but, as every scientific approach, with important limitations. Indeed, cell-cell and cell-tissue interactions are often complicated to study in live animals and require advanced technologies and long experimental timing. In this field, zebrafish (ZF) partially overcomes these limitations. The transparency of ZF embryos and the high number of available engineered cell lines make this model particularly suitable for innovative imaging techniques up to the visualization of single cell processes (41). For instance, transgenic strains characterized by the expression of fluorescent proteins in specific cell populations allow the study of their crosstalk with cancer components with more accuracy. Among them, engineered ZF presenting green fluorescent neutrophils (Tg(mpx:eGFP)) or characterized by macrophages expressing different fluorophores depending on their polarization status (Tg(mpeg1:mCherry-F; tnfa:eGFP-F)) were used to study the interaction between colorectal cancer and immune cells at the level of physical cell interactions (42). The possibility to visualize and track cancer cells in the transparent body of ZF embryos represents an ideal feature for the study of the crosstalk between metastatic and endothelial cells. Besides the simple evaluation of the dissemination properties, transgenic ZF models with fluorescent blood vessels can be employed also to test the influence of physical cues and biomechanical stimuli on the interaction of cancer cells with endothelium and distant tissues. In this field, Follain et al. demonstrated the implication of the hemodynamic forces in the metastatic properties of circulating tumor cells through the study of ZF blood flow profiles while Paul et al. used the vessel topography analysis to identify tissue architectural cues driving organ selectivity cell-type-dependent extravasation (43, 44). Murine model still represent the most widely used mammalian in cancer research representing an indispensable platform for the de velopment of novel methods of prevention, diagnosis, and treatment before going into clinical trial (31, 45). Humanized murine models established transplanting human-derived hematopoietic stem cells (HSCs) or peripheral blood mononuclear cells (PBMCs) into immunodeficient mice can be used as a system in which human tumors grown in the presence of a competent human immune system (46). This model allows studying immune-tumor crosstalk also determining the different immune cells that infiltrate the TME. In addition, this model has been extensively used to test a wide variety of immunotherapeutic approaches including adoptive cell therapies (47), treatment with specific antibodies (48) and also oncolytic viruses (49). Nevertheless, advances in the development of humanized mouse models are still need to increase their translational potential.

## 5 Future perspectives

The continuous increasing of cancer characterization requires systems even more complex. Each model is designed to reproduce only some of the pathophysiological aspects of the tumor disease and the development of a realistic “tumor organ” is still impossible by a single model. To overcome these limitations, researchers integrate different systems to increase their fields of applications and the number of variables to take in consideration (**Figure 2**). 3D cultures have gradually replaced the common flat supports but the static TME condition continue to represent an experimental limit. The application of these models in microfluidic devices allows the connection of cancer cultures to distant biological elements through the dynamic media circulation. Although the addition of fluidic structure, the influence of blood cells, vessels mechanic stimuli and tissue secretions remain processes reproducible only in a complete organism. In this context, the transplantation of 3D cultures in ZF or GEMMs can be a promising future tool to study the behaviors of controllable tumor systems in a live microenvironment. The models integration represents one possibility but, in the era of omic data, the sequencing analysis could be used to improve the *in vitro* and *in vivo* approaches. Indeed, the design of algorithms using the clinic and molecular patients profiles combine the increasing of data reliability and the development of personalized models. These aspects acquire particular relevance in the preclinical studies that needed patients-based tools to perform drug screening. In conclusion, we have discussed and supposed different kind of approaches to reach the real tumor complexity. The main aim is to supply an overview of the most innovative model integration strategies in support of the concept that only through the gradual addition of controllable variables the researcher can create a realistic “tumor organ”.

## Data availability statement

The original contributions presented in the study are included in the article/supplementary material. Further inquiries can be directed to the corresponding authors.

## Author contributions

All authors listed have made a substantial, direct, and intellectual contribution to the work, and approved it for publication.
